# Reports of Meetings

**Published:** 1952-07

**Authors:** 


### Reports of Meetings

### DEVON AND EXETER MEDICO-CHIRURGICAL SOCIETY

A meeting was held at Hawkmoor Chest Hospital, Bovey Tracey, Devon, on April 17th, 1952. After an inspection of the hospital Dr. R. L. Midgley gave a short account of some cases which he was treating with *Nydrazid*, the Squibb's isonicotinic acid hydrazide preparation. Some twenty cases were reviewed, but as the average length of time they had been under treatment was only four weeks, nothing more than a tentative opinion could be expressed. There had been no untoward effects, and the results so far obtained were sufficiently encouraging to warrant further investigation. Mr. J. L. Griffith gave a most interesting demonstration of cases and pathological specimens to illustrate the place of major surgery in the treatment of pulmonary tuberculosis and of non-tuberculous thoracic disease.

### SURGICAL CLUB OF SOUTH WEST ENGLAND

The seventh post-war meeting of the *Surgical Club of South West England* was held at the Royal Cornwall Infirmary, Truro, on May 2nd and 3rd, 1952.

On the Friday morning demonstration operations were performed by Mr. T. E. Rutter (Truro), Mr. S. R. Adlington (Falmouth) and Mr. C. B. O'Carroll (Falmouth), after which specimens and X-rays were discussed until lunch-time. During the afternoon four papers were read on: *Tantalum gauze implant in the treatment of inguinal hernia*, by Mr. G. F. Burnell (Truro); *One hundred cases of gall-bladder diseases*, Mr. J. R. White (Penzance); *Acute gastric haemorrhage*, Mr. T. Reid (Camborne–Redruth), and *The management of carcinoma of the colon*, Mr. S. R. Adlington (Falmouth).

In the evening a cocktail party was given by the local surgeons at the Goonvrea Hotel and this was followed by the official dinner at Truro.

On Saturday morning more operations were performed by Mr. T. Reid and Mr. J. R. White; afterwards papers were read by Mr. T. E. Rutter on *Carcinoma of the bladder*, and Mr. C. B. O'Carroll on *Acute intestinal obstruction in the aged patient*, both of which were followed by a lively discussion.

The meeting was notable not only for the excellence of the official programme, but also for the beautiful spring weather and the two lunches kindly arranged by the West Cornwall Hospital Management Committee in the Nurses' Home of the Royal Cornwall Infirmary.

### SOUTH WEST ORTHOPAEDIC CLUB

The Spring meeting of the *South West Orthopaedic Club* was held at Musgrove Park Hospital, Taunton, on Saturday, May 24th, 1952. Mr. R. F. Winckworth was Chairman.

Mr. K. Pridie showed serial sections of the heads of several femora which had been excised for avascular necrosis following fracture of the neck of the femur. He emphasized the importance of the blood supply of the head of the femur, particularly that of the ligamentum teres and its anastomosis with the branches of the circumflex vessels. He said that when non-union occurred following the out-of-date method of treatment by doing nothing except to encourage active exercises of the hip, the head of the femur never disintegrated, but became revascularized. He felt that weight bearing was responsible for the fragmentation which occurred with avascular necrosis after pinning.

Mr. J. Bastow said he thought this evidence showed how unwise it was to allow early weight bearing after pinning, and that a reasonable time for union should be allowed, say six months or more, before permitting the patient to take weight. The X-ray was of little value in the determination of union.

Mr. A. EYRE BROOK showed some arthrograms of cases of congenital dislocation of the hip. The arthrograms had been carried out after the period of immobilization had been terminated. He felt that little could be gained from an arthrogram carried out before the reduction was effected, but there was much to be seen in the post-fixation period. It outlined the limbus clearly and gave assistance in deciding if a shelf operation was necessary. It showed up the interior of the joint and disclosed any obstruction in the acetabulum. Some cases appeared to show recurrent subluxation of the head of the femur on a straight X-ray film, but, in fact, were not doing so when visualized on the arthrogram.

He had carried out an arthrogram on an old case of Perthe's disease of the hip and the interesting fact was that there was congruity of the joint surfaces although they were deformed.

He also showed a case of subluxation of the hip which had occurred following anterior poliomyelitis. Although rotation was said to cause the dislocation, yet in this instance rotation one way or the other did not appear to make any difference in the arthrogram. He had carried out an external rotation osteotomy on this case with no improvement.

Mr. F. C. DURBIN said that he felt that the early arthrograms should always be done in order to distinguish between the true luxation and primary subluxation of Leveuf. This might have an important bearing on progress.

Later on, when the ossification of the head of the femur was eccentric, an appearance of subluxation could be produced which was shown to be false by an arthrogram.

Dr. M. MCALLEY said that during the war he had treated about 100 cases of traumatic myositis ossificans with deep X-rays. Most of the cases occurred in the army; in many of them the condition was found in the region of the elbow joint. They were given 1,500 V. in divided doses at 140 K.V. and at weekly intervals for six weeks. No treatment other than the X-rays was given and the patient was allowed activity of the involved muscles. He said that pain disappeared in three weeks and did not recur: the new bone formation became less in extent, but did not always vanish.

Mr. BLUNDELL JONES discussed eight cases of paralytic spontaneous dislocation of the hip occurring in infancy in both cerebral palsy and poliomyelitis. He considered that valgus deformity of the femoral neck was a primary factor in the production of dislocation and that this could cause luxation even without adduction muscle forces. Valgus of the femoral neck occurred where rapid growth was released from the moulding forces of muscle action and weight bearing, so that the neck continued to grow in line with the shaft. It was therefore most marked in cases paralysed during the first two years of life. Two bilateral cases were presented in which good stability of the hip had been restored by osteotomy at the base of the femoral neck. In one of these, a miniature Capener Neufeld nail plate had been used for fixation. Osteotomy should be carried out as soon as subluxation was recognized in order to prevent secondary growth deformity of the acetabulum; in the later stages, only a shelf stabilization would answer since the acetabulum becomes filled up and shallow.

In discussing a case of paralytic dislocation in Mr. Eyre Brook's series, Mr. BLUNDELL JONES pointed out that stability had been achieved not as the result of an intended rotation osteotomy, but because the operation had also corrected the deformity of the femoral neck. Anteversion had not been a factor in any of his cases.

Mr. KEITH LUCAS said that he had followed up some thirty cases of pathological dislocation of the hips at Liverpool, some of which had been treated initially by Sir Robert Jones. He said that although many operative procedures had been carried out, yet in every case the dislocation had recurred. In no case had an osteotomy followed by internal fixation been done. This might be the solution to a difficult problem.

Mr. F. C. DURBIN showed slides of nine cases of subcapital fractures of the neck of the femur, treated with Austin Moore pins. There was some statistical evidence (Linton 1944) to suggest that the large bulk of the ordinary Smith Petersen nail increased the incidence of avascular necrosis due to further damage to the blood supply of the head. This, it had been suggested (Tucker 1949), might be avoided by the use of multiple small pins.

Of the nine cases, there was evidence of avascular necrosis in six, and it was complete in three cases. All except two cases had a fracture shaft angle less than 30 deg. The results were not sufficiently encouraging to justify a continuation of the method.

There were other difficulties:

1. Multiple pins were more difficult to insert accurately, and the operation time was longer in comparison with a Smith Petersen pin.
2. It was difficult to impact the fracture with the pins in position.
3. The pins sometimes penetrated the joint.
4. In two cases the pins fractured.

He felt that there was a place for the use of multiple pins in the fixation of the slipped upper femoral epiphysis.

When the epiphysis had rotated backwards through one-third of its circumference, it was quite easy to obtain a good hold on it with multiple pins, but very difficult to do so with a single Smith Petersen pin.

It was also quite easy to insert the pins when the epiphysis was hard, and it possibly involved less trauma.

When extracting the pins, they had to be unscrewed. Unless the points had the shoulders rounded off, they were locked by bone and unscrewing them led to fracture of the pins.

Mr. Robins showed some colour slides illustrating the treatment of subcutaneous infections of the hand, complicated by skin necrosis. He emphasized the importance of operating in a bloodless field and of draining the abscess through the area of devitalized skin. A granulating area remaining after the infection has been controlled, should be covered with a skin graft.

Mr. Tom Price was unable to be present owing to illness, and a paper of his reviewing the literature and describing a case of congenital flat foot, was read for him by Mr. Hedly Hall. The deformity was difficult to treat as it nearly always relapsed.

Mr. Durbin showed X-ray pictures of a mild case of congenital flat foot which had not been treated. Twenty years later, the patient had a good, painless, functioning foot. A recent X-ray showed that a pseudarthrosis had occurred between the navicular and the dorsum of the talus.

Clinical cases were demonstrated at the afternoon session by Mr. Jowett and Mr. Winckworth. Among many cases of great clinical interest were the closure of cavities in bone by muscle flaps and chip bone grafts, and a case of tuberculosis of the patella.

### SOUTH WEST REGIONAL PAEDIATRIC CLUB

The *South West Regional Paediatric Club* met at Taunton on May 17th and 18th. Cases were shown and discussed by Dr. J. Coates and others, and the following papers were read:

Dr. A. H. Banton: **"Mediterranean Anaemia."**
Dr. Davidson: **"Twenty-five Years of Experience in Child Health."**
Dr. H. R. Jolly: **"Sexual Precocity."**

### SYMPOSIUM ON THE SUPRARENAL CORTEX

The Colston Research Society, which for many years has made grants in aid of research in the University of Bristol, has for some years arranged annual symposia on selected research topics of current importance. Four of these symposia have been held on: Cosmic Radiation, Engineering Structures, Colonial Administration and The Universities and the Theatre. The Fifth Colston Symposium, on The Suprarenal Cortex, was held on April 1st, 2nd and 3rd. The scientific sessions were held in the Physics Lecture Theatre, and most of the members were accommodated at a nearby University Hall of Residence, Manor Hall. The organization was in the competent hands of Professor Yoffey.

There were 136 members altogether including fourteen guest speakers. Among the visitors was a strong contingent from the United States, comprising Drs. Li, Astwood, Ingle, Robinson and Hoagland. In addition, there were visitors from France, Belgium,

Holland, Denmark, Portugal, Madeira, Switzerland and Germany. Furthermore, a number of " locals ", averaging about thirty per session, attended the symposium.

The full proceedings of the symposium will be published in a special Symposium Volume, and it would not serve any useful purpose to give a very brief and incomplete summary of the proceedings.

The broad plan was prepared so as to commence the proceedings with a consideration of the mechanism for controlling the activity of the suprarenal cortex. This part of the symposium centred mainly around *ACTH* and its action. In the second group of contributions were considered the nature of the cortical secretion and its action upon various parts of the body. Finally, there was a clinical session in which the surgical and medical applications of the various basic data were discussed.

The symposium on the whole passed off very smoothly, and the Physics Lecture Theatre proved to be an admirable hall for the meetings. The breaks for coffee and light refreshments between the sessions provided ample opportunity for the rather more vigorous and unrestrained discussion which constitutes so valuable a feature of these symposia.

Overseas visitors seemed to be particularly impressed by the fact that adequate time was allowed for the discussion, and at none of the sessions were members who wished to participate in the discussion precluded from so doing.

### Programme of Discussions

1. THE PREPARATION AND ASSAY OF *ACTH*
Dr. C. H. Li, University of California, U.S.A.

2. THE PHYSICAL AND CHEMICAL PROPERTIES OF *ACTH*
Professor F. G. Young, Cambridge University.

3. THE PURIFICATION OF *ACTH*
Dr. C. J. O. R. Morris, The London Hospital, London.

4. THE SUPRARENAL CORTEX, THE STRUCTURAL BACKGROUND
Professor J. M. Yoffey, University of Bristol.

5. THE NATURE OF THE ADRENAL CORTICAL SECRETION
Professor F. Verzar, University of Basel, Switzerland.

6. CONTROL OF THE SECRETORY ACTIVITY OF THE SUPRARENAL CORTEX, WITH SPECIAL REFERENCE TO THE ISOLATED PREPARATION
Dr. Marthe Vogt, University of Edinburgh.

7. THE SUPRARENAL CORTEX AND THE GONADS
Professor S. Zuckerman, University of Birmingham.

8. THE METABOLISM OF THE ADRENO-CORTICAL STEROIDS
Professor G. F. Marrian, University of Edinburgh.

9. THE ROLE OF THE ADRENAL GLANDS IN INFECTION AND INTOXICATION
Dr. Harry J. Robinson, The Merck Institute, Rahway, U.S.A.

10. ADRENAL STEROIDS AND PERSONALITY DISORDERS
Dr. Hudson Hoagland, The Worcester Foundation, Shrewsbury, Mass., U.S.A.

11. CHANGES IN SUPRARENAL CORTEX FUNCTION IN SHOCK AND HORMONE TREATMENTS
Dr. R. E. Hemphill, Bristol Mental Hospital.

12. SUPRARENAL CORTEX ACTIVITY IN THE ENDOCRINE EQUILIBRIUM OF HUMANS

Dr. M. Reiss, Bristol Mental Hospital.

13. *ACTH*, STEROID HORMONES AND TISSUE CHANGES
Professor G. R. CAMERON, University College Hospital Medical School, London.

14. THE ROLE OF THE ADRENAL CORTEX IN HOMEOSTASIS
Dr. DWIGHT J. INGLE, Upjohn Company, Mich., U.S.A.

15. THE INFLUENCE OF THE SUPRARENAL CORTEX ON MINERAL AND WATER METABOLISM
Professor H. HELLER, University of Bristol.

16. THE SURGERY OF THE SUPRARENAL GLAND WITH ESPECIAL REFERENCE TO THE CORTEX
Mr. L. R. BROSTER, Charing Cross Hospital, London.

17. CLINICAL APPLICATIONS OF *ACTH* AND STEROID HORMONES
Professor GEORGE W. THORN, Harvard Medical School, Boston, Mass., U.S.A.

18. THE CLINICAL RESPONSES IN THE RHEUMATIC DISEASES
Dr. G. D. KERSLEY, Royal National Hospital for Rheumatic Diseases, Bath.

